# Identification through machine learning of potential immune- related gene biomarkers associated with immune cell infiltration in myocardial infarction

**DOI:** 10.1186/s12872-023-03196-w

**Published:** 2023-03-28

**Authors:** Hao Dong, Shi-Bai Yan, Guo-Sheng Li, Zhi-Guang Huang, Dong-Ming Li, Yu-lu Tang, Jia-Qian Le, Yan-Fang Pan, Zhen Yang, Hong-Bo Pan, Gang Chen, Ming-Jie Li

**Affiliations:** 1 Department of Cardiovascular Medicine, The First Affiliated Hospital of Guangxi Medical University, No.6 Shuangyong Road, Nanning, Guangxi Zhuang Autonomous Region 530021 People’s Republic of China; 2 Department of Pathology/ Forensic Medicine, The First Affiliated Hospital of Guangxi Medical University, No.6 Shuangyong Road, Nanning, Guangxi Zhuang Autonomous Region 530021 People’s Republic of China; 3 Department of Cardiothoracic Surgery, The First Affiliated Hospital of Guangxi Medical University, No.6 Shuangyong Road, Nanning, Guangxi Zhuang Autonomous Region 530021 People’s Republic of China; 4 Department of Pathology, Hospital of Guangxi Liugang Medical Co.LTD./Guangxi Liuzhou Dingshun Forensic Expert Institute, No.9, Queershan Rd, Liuzhou, Guangxi Zhuang Autonomous Region 545002 People’s Republic of China; 5 Department of Gerontology, NO.923 Hospital of Chinese People’s Liberation Army, No. 1 Tangcheng Rd, Nanning, Guangxi Zhuang Autonomous Region 530021 People’s Republic of China

**Keywords:** Immune-related gene, Immune cell infiltration, CIBERSORT, Nomogram

## Abstract

**Background:**

To investigate the potential role of immune-related genes (IRGs) and immune cells in myocardial infarction (MI) and establish a nomogram model for diagnosing myocardial infarction.

**Methods:**

Raw and processed gene expression profiling datasets were archived from the Gene Expression Omnibus (GEO) database. Differentially expressed immune-related genes (DIRGs), which were screened out by four machine learning algorithms-partial least squares (PLS), random forest model (RF), k-nearest neighbor (KNN), and support vector machine model (SVM) were used in the diagnosis of MI.

**Results:**

The six key DIRGs (PTGER2, LGR6, IL17B, IL13RA1, CCL4, and ADM) were identified by the intersection of the minimal root mean square error (RMSE) of four machine learning algorithms, which were screened out to establish the nomogram model to predict the incidence of MI by using the rms package. The nomogram model exhibited the highest predictive accuracy and better potential clinical utility. The relative distribution of 22 types of immune cells was evaluated using cell type identification, which was done by estimating relative subsets of RNA transcripts (CIBERSORT) algorithm. The distribution of four types of immune cells, such as plasma cells, T cells follicular helper, Mast cells resting, and neutrophils, was significantly upregulated in MI, while five types of immune cell dispersion, T cells CD4 naive, macrophages M1, macrophages M2, dendritic cells resting, and mast cells activated in MI patients, were significantly downregulated in MI.

**Conclusion:**

This study demonstrated that IRGs were correlated with MI, suggesting that immune cells may be potential therapeutic targets of immunotherapy in MI.

## Introduction

The mortality of coronary artery disease (CAD) has decreased in recent decades, but it remains the main cause of mortality worldwide [1]. For example, over the past 35 years, 1.7 million deaths annually have been attributed to CAD in the USA and Europe [2]. One of the biggest causes of mortality in CAD is MI [1]. With the accessibility of coronary interventions, coronary bypass surgery, and drugs, early diagnosis and risk stratification can significantly reduce mortality in MI. Traditional biomarkers in the early diagnosis of MI, such as high-sensitivity cardiac troponin T (hs-cTnT), high-sensitivity cardiac troponin I (hs-cTnI), and creatine kinase-MB, have been demonstrated with high sensitivity but without specificity [3]. As a result, there is a need to screen out the novel diagnostic biomarkers of MI.

Patients with CAD can be classified as either chronic coronary syndromes (CCS) or acute coronary syndromes (ACS), depending on the clinical symptoms the patient is presenting [4]. The literature has demonstrated that the conversion from CCS to ACS is typically initiated by an acute atherothrombotic event, which results in the rupture or erosion of atherosclerotic plaques [5]. Many conventional risk factors, such as smoking, high cholesterol, obesity, diabetes mellitus, and hypertension, are responsible for the incidence of CAD by participating in the immune microenvironment [6]. However, there is less evidence suggesting a relationship between the pathogenesis of MI and immune genes or immune cells or inflammatory mediators.

With the advancement of microarray analysis, an increasing number of studies have demonstrated that genes can be targeted for early diagnosis, classification, prognosis, prediction of disease severity, and new drugs. For example, several genes in peripheral blood mononuclear cells, such as ADAP2, KLRC1, MIR21, PDGFD, and CD14, were demonstrated as having a significant signature for categorizing MI patients and normal controls [7]. ASCC2, LRRC18, and SLC25A37 have not only been demonstrated as the diagnostic biomarkers of CAD, but also have closely participated in the pathogenesis and advancement of CAD [8]. There is little research that has analyzed the immune genes of CAD and MI.

Many pieces of research have indicated that immune cell infiltration is closely related to the onset of MI. For instance, increased apoptosis of lymphocytes in peripheral blood and infiltrated proinflammatory Th1 lymphocytes was observed in pig hearts after reperfusion within 48 h, and circulating T lymphocytes were significantly decreased in post-PCI MI patients within the first 24 h [9]. Cell type identification by estimating the relative subsets of RNA transcripts (CIBERSORT) has been widely implemented to portray immune cell ratios from RNA-seq data of samples from various diseases [10]. However, there is little research on immune cell infiltration analysis in MI patients conducted using CIBERSORT.

In our study, the raw gene expression profiling datasets of MI patients and normal controls were archived from the GEO database, and the intersections between IRGs and differentially expressed genes (DEGs) were identified for further analysis. Diagnostic biomarkers were identified using four machine learning algorithms. Subsequently, the relative proportion of 22 different types of immune cells in patients with MI and in normal controls was calculated using CIBERSORT. Finally, the potential role of diagnostic IRGs associated with immune cell infiltration was verified in patients with MI using machine learning.

## Material and methods

### Microarray data source

**Fig. 1:**
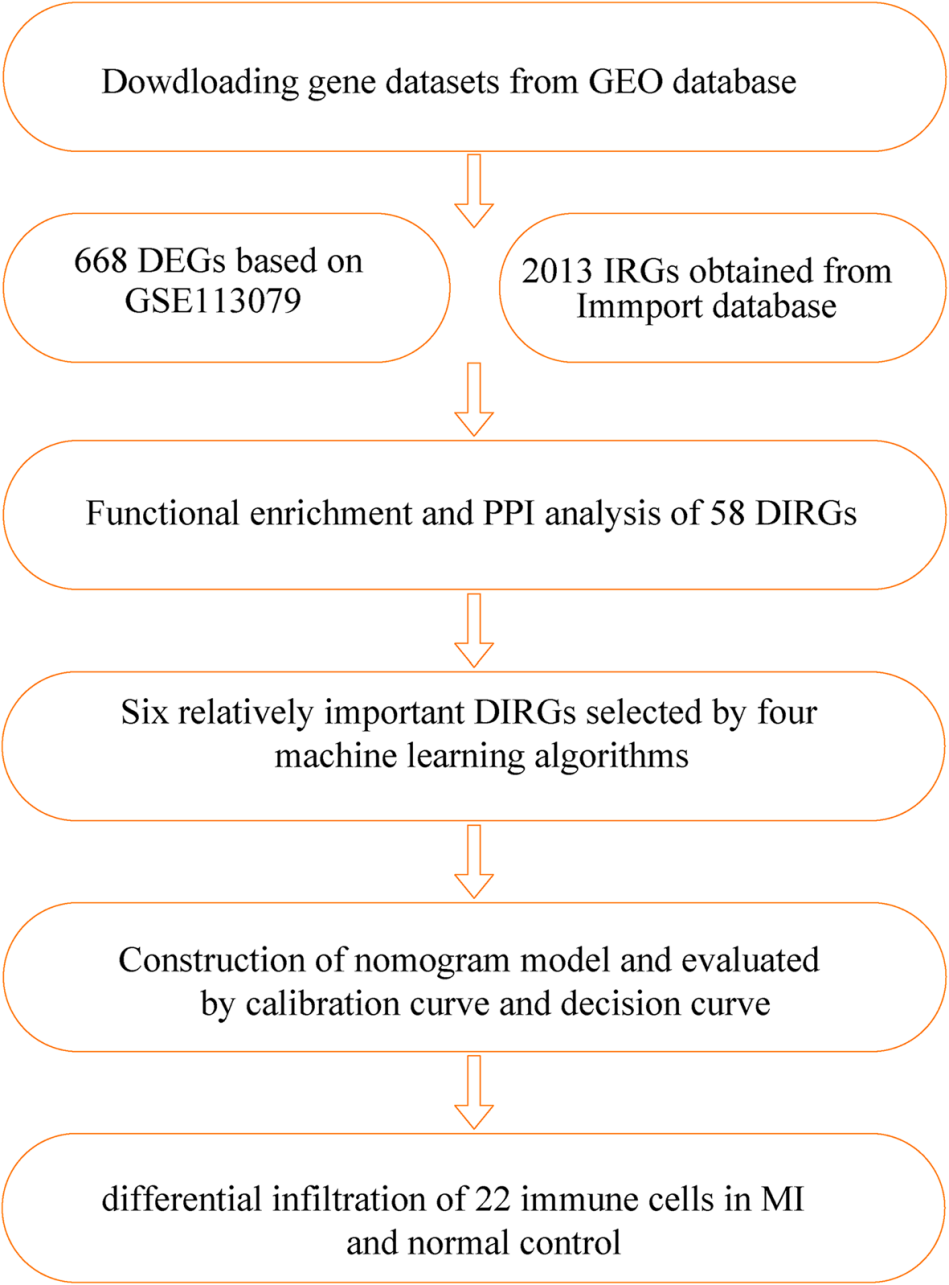
Schematic diagram of study

The GEO database (http://www.ncbi.nlm.nih.gov/geo) was explored to download the datasets according to keywords such as “coronary artery disease” or “Myocardial infarction.” The gene dataset was considered eligible according to the following inclusion criteria: (1) datasets belonging to humans and (2) the source of tissue was blood. The exclusion criteria were as follows: (1) duplicated datasets, (2) without case control data, and (3) nonhuman data. The GSE113079 contains 97 CAD patients and healthy controls. A new data cohort merged by four GEO datasets (GSE29111, GSE48060, GSE66360, and GSE97320) contains 101 MI patients and 74 healthy controls. The information of five GEO datasets (GSE29111, GSE48060, GSE66360, GSE97320, and GSE113079) are included in Table 1. Here, 2,484 IRGs were archived from ImmPort (https://www.immport.org) [11]. The schematic diagram of our study is shown in Fig. 1.

**Table 1:** Information of the involved gene datasets

ID	Year	Platform	Region	Tissue	Sample
GSE29111	2011	GPL570	United Kingdom	Peripheral blood	N:MI = 0:18
GSE48060	2014	GPL570	USA	Peripheral blood	N:MI = 21:31
GSE66360	2015	GPL570	USA	Peripheral blood	N:MI = 50:49
GSE97320	2017	GPL570	China	Peripheral blood	N:MI = 3:3
GSE113079	2018	GPL20115	China	Peripheral blood	N:CAD = 48:93

*Abbreviations*: *N* Normal control, *MI* Myocardial infarction, *CAD* coronary artery disease

### Data processing and differentially expressed gene analysis

**Fig. 2:**
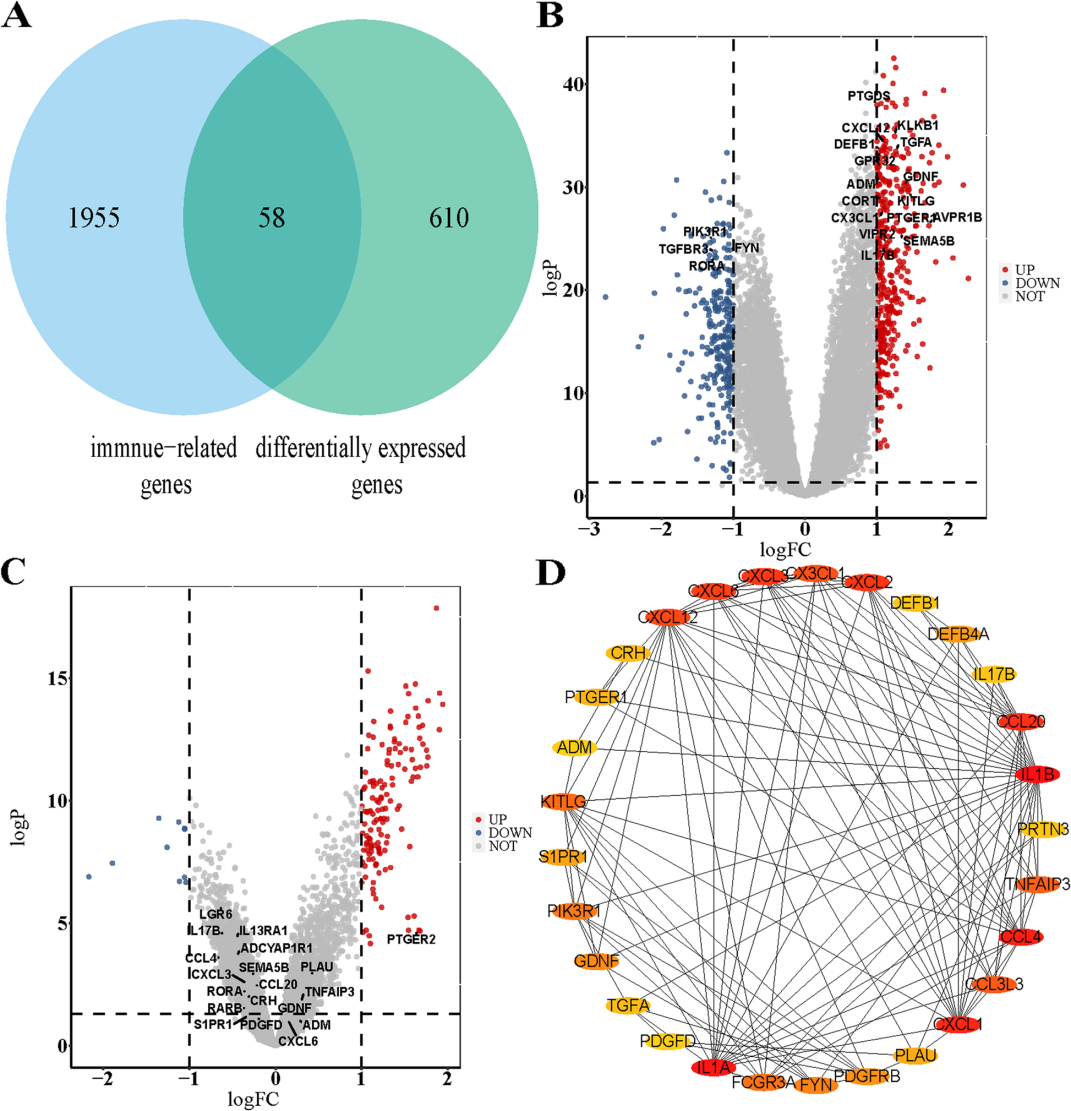
Differentially expressed gene analysis and protein–protein interaction networks. **A** Intersection of 668 DEGs and 2013 IRGs. **B** volcano plot in GSE113079. **C** volcano plot in merged GP570 datasets. **D** The PPI network of the 58 DIRGs

The DEGs with a threshold of a *P* value of < 0.05 and log(fold change) > 1 or log(fold change) < -1 between patients with CAD and normal controls in GSE113079 were obtained by the limma package [12]. DIRGs were obtained by overlapping DEGs and IRGs, as shown in Fig. 2. The relationship between DIRGs and the incidence of MI was verified using a new data cohort. First, the “RMA” function of the Affy package was applied to raw data in the new data cohort for background correction and quantile normalization [13], and the batch effect was removed by the “removeBatchEffect” function in the sva package [14]. The DEGs between MI patients and normal controls were screened by conducting the limma package. The volcano plot of DEGs was visualized by the ggplot2 package.

### Functional enrichment analysis of DIRGs

To explore the mechanism of the incidence of MI, gene ontology (GO) and Kyoto Encyclopedia of Genes and Genomes (KEGG) pathway enrichment [15–17] were performed to annotate the genes and gene products. GO analysis was conducted to identify the biological process (BP), cellular components (CC), and molecular function (MF) of key DIRGs. KEGG, consisting of chemical and systemic functional information, was implemented to identify functional and metabolic pathways. DO (disease ontology) analysis was conducted to combine biomedical data with human disease. Metascape (www.metascape.org) is an online website that provides a comprehensive gene annotation and analysis resource [18]. DIRGs were imported to Metascape to analyze the GO and KEGG pathway analysis, and the DOSE package was conducted to investigate the DO analysis of DIRGs [19].

### Integration of PPI network to select hub genes

The STRING database (http://string-db.org/) aims to investigate protein interactions, both known and predicted. The protein–protein interaction network (PPI) of DIRGs was constructed by the STRING database. Cytoscape is one open-source software tool for bioinformatics analysis, which is conducted for PPI network visualization and analysis. The key modules of the PPI network, here identified by the molecular complex detection (MCODE) plug-in of Cytoscape software, were widely implemented to screen out significant parts according to the default parameter settings (Degree Cutoff = 2, Node Score Cutoff = 0. 2, K-Core = 2,and Max Depth = 100).

### Key DIRGs selected by the PLS, GLM, and SVM algorithms

To develop the diagnosis model for patients with MI, we conducted four machine learning algorithms,such as partial least squares (PLS), random forest model (RF), k-nearest neighbor (KNN), and support vector machine model (SVM).PLS is a multivariate statistical data analysis,considered as the combination of Principal Component Analysys and multiple linear regression analysis, which could develop accurate prediction model when variables significantly correlated [20]. RF is a learning method for classification,regression and other tasks,which builds decision trees on different samples and scores the classification results, and RF model will use statistical analysis on the classification results to screen out high accuracy classification results of all single trees [21]. SVM was a supervised machine-learning technique for classification and regression. It can filter out the feature subset of the highest accuracy results in a large amount of data [22]. KNN is a non-parametric, supervised learning classifier which uses proximity to make classifications about the grouping of an individual data point to find a predetermined number of training samples closest in the distance to a new point and provide a value for the data [23]. PLS, KNN, RF, and SVM were constructed in a new data cohort merged by four GEO datasets (GSE29111, GSE48060, GSE66360, and GSE97320) by the DALEX package in R. Significant DIRGs were used as explanatory features to distinguish MI patients and normal controls in a new data cohort. The residual distribution was shown to have the best model with minimal residuals, and the importance of the model in DIRGs was selected by the root mean square error (RMSE). Finally, the six most important DIRGs were selected by the above four models for further study.

### A nomogram model constructed and assessed for a diagnosis of MI

A nomogram model consisting of six significant DIRGs was constructed using the rms package for predicting the incidence of MI. “Points” were demonstrated separately as the score of the six most important DIRGs, and “Total Points” was the summation of the above DIRGs. The Area Under Curve (AUC) was implemented to assess the discrimination ability of nomogram model and each DIRGs. A calibration curve was constructed to plot the predictive and actual probability of the nomogram model. Finally, the clinical usefulness and effectiveness of the nomogram model were demonstrated by decision curve analysis.

### Distribution of immune cells

As a computational deconvolution algorithm method, CIBERSORT can characterize the cell composition of complex tissue from gene expression profiles. Twenty-two immune cell types in a new data cohort were calculated by CIBERSORT. We then compared the distribution of 22 types of immune cells between patients with MI and normal controls.

### The association between key DIRGs and the immune cell infiltration of MI

Pearson correlation analysis was implemented to estimate the association between key DIRGs and immune cell infiltration, here by using the psych package and as visualized by the ggplot2 package.

## Results

### Data processing and differentially expressed gene analysis

According to the following criterion of a *P* value < 0.05 and log (fold change) > 1 or log(fold change) < -1,668, DEGs between patients with CAD and normal controls were archived in the GSE113079 dataset by the limma package. To determine the significant IRGs from the DEGs, 58 DIRGs were obtained after the intersection between 668 DEGs and 2013 IRG (Fig. 2A). Here, 58 DIRGs were shown by volcano plot in GSE113079 and the new dataset (Fig. 2B, C). The PPI network of the 58 DIRGs was investigated using STRING 11.0 and visualizing in Cytoscape 3.8.0 (Fig. 2D).

### GO, KEGG, and DO pathway enrichment analysis

**Fig. 3:**
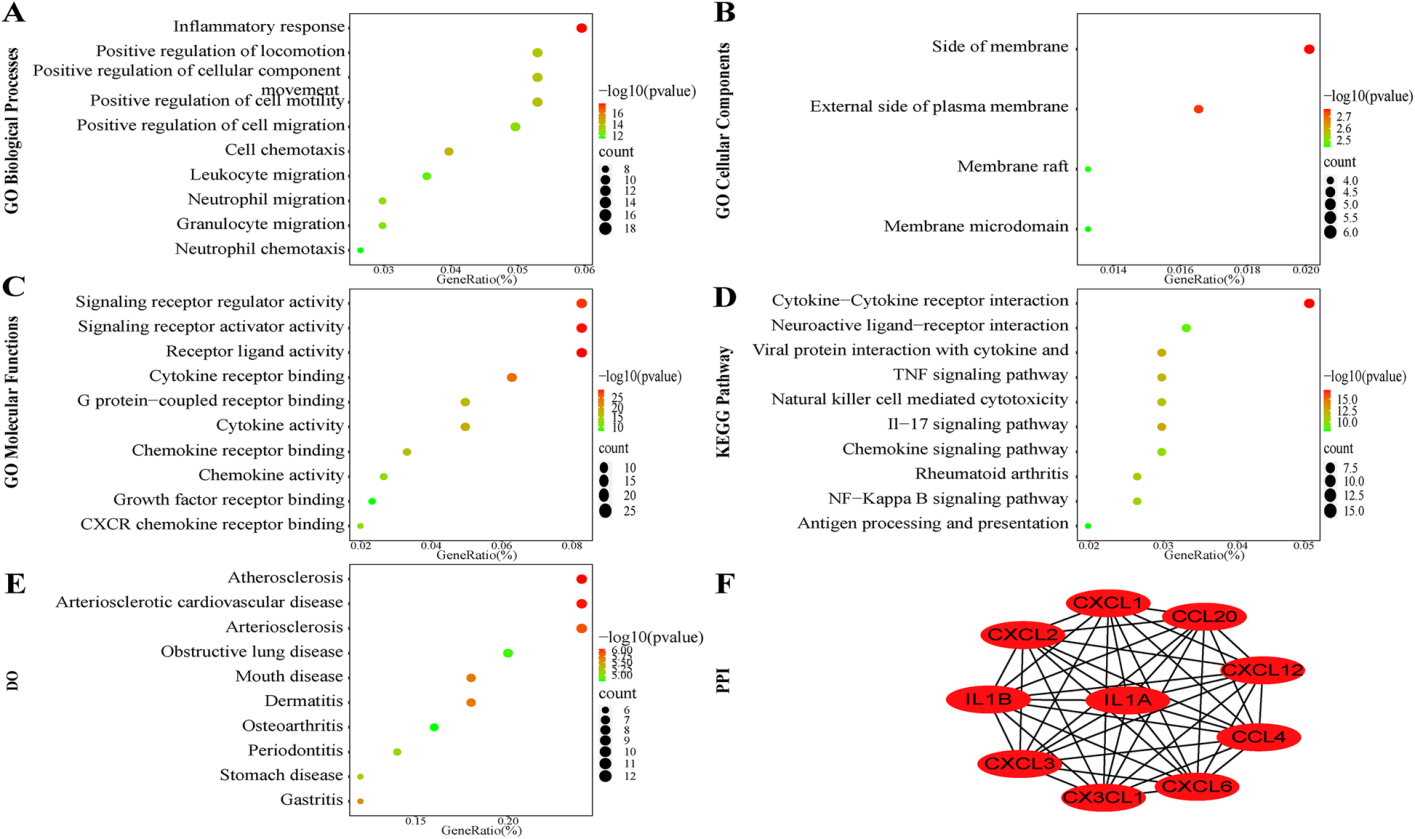
GO, KEGG, and DO pathway enrichment analysis. **A** BP of 58 DIRGs. **B** CC of 58 DIRGs. **C**: MF of 58 DIRGs. **D** KEGG pathway of 58 DIRGs. **E** DO pathway of 58 DIRGs. **F** PPI of hub genes

Fifty-eight DIRGs were imported to the Metascape website to explore the enrichment analysis visualized by the ggplot2 package. GO analysis, as explored by the Metascape online database, was conducted to cluster the functions of the BP, CC, and MF. The results demonstrated that 58 DIRGs were enriched in the inflammatory response of BP, side of the membrane of CC, and signaling receptor regulator activity of MF (Fig. 3A, B, C). The KEGG pathway showed that 58 DIRGs were mostly enriched in cytokine–cytokine receptor interactions (Fig. 3D). The DO pathway analysis was mainly enriched in atherosclerosis (Fig. 3E). These results indicated that inflammation may be significantly associated with the incidence of CAD.

### Analysis of the PPI network

A PPI network of 58 DIRGs was utilized on the STRING website and visualized by Cytoscape. MCODE plug-ins were performed to screen out the most vital function modules in the total protein–protein networks. Significant modules consisted of key genes with 10 nodes and 90 edges, including IL1B, IL1A, CXCL1, CXCL2, CXCL6, CXCL3, CXCL12, CX3CL1, CCL4, and CCL20 (Fig. 3F).

### Significant DIRGs selected by PLS, GLM, and SVM algorithms

**Fig. 4:**
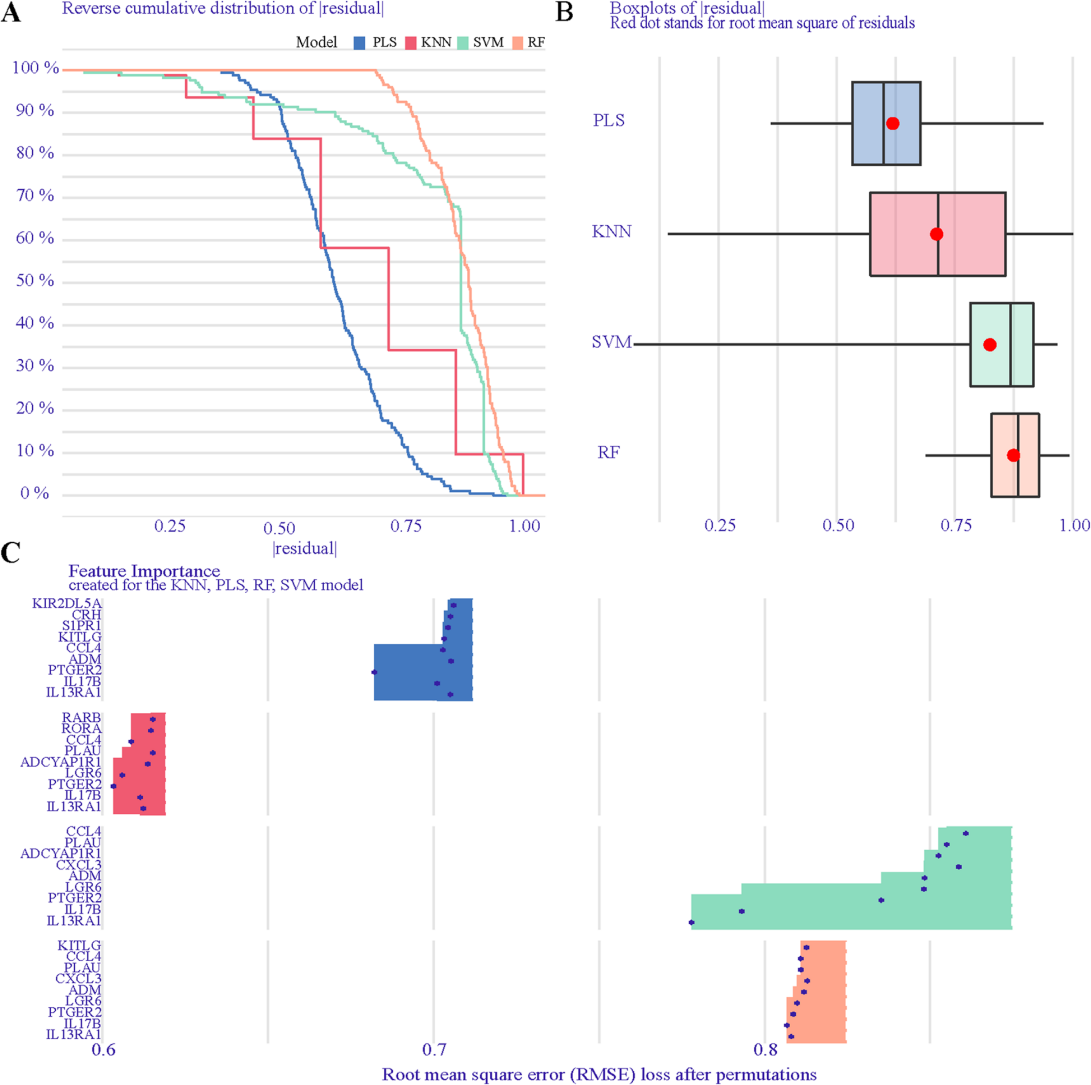
Significant DIRGs of the RF, PLS, SVM, and KNN models. **A** Cumulative residual distribution of the RF, PLS, SVM, and KNN models. **B** Boxplots of the residuals in the RF, PLS, SVM, and KNN models. **C** The importance of significant DIRGs in the RF, PLS, SVM, and KNN models

To investigate the connection between key IRGs and the prevalence of MI, PLS, RF, KNN, and SVM were independently constructed to narrow down key IRGs using the new data cohort. The importance of explanatory features and residual distribution of four models were analyzed and visualized by the DALEX package in the new data cohort. The PLS model was demonstrated to be the best suitable model with the lowest residual (Fig. 4A and B). The importance of 58 DIRGs based on the above four models is shown in Fig. 4C. The intersection of six genes in the four models of minimal RMSE was PTGER2, LGR6, IL17B, IL13RA1, CCL4, and ADM. These six genes were applied for further analysis.

### Further analysis of six DIRGs

**Fig. 5:**
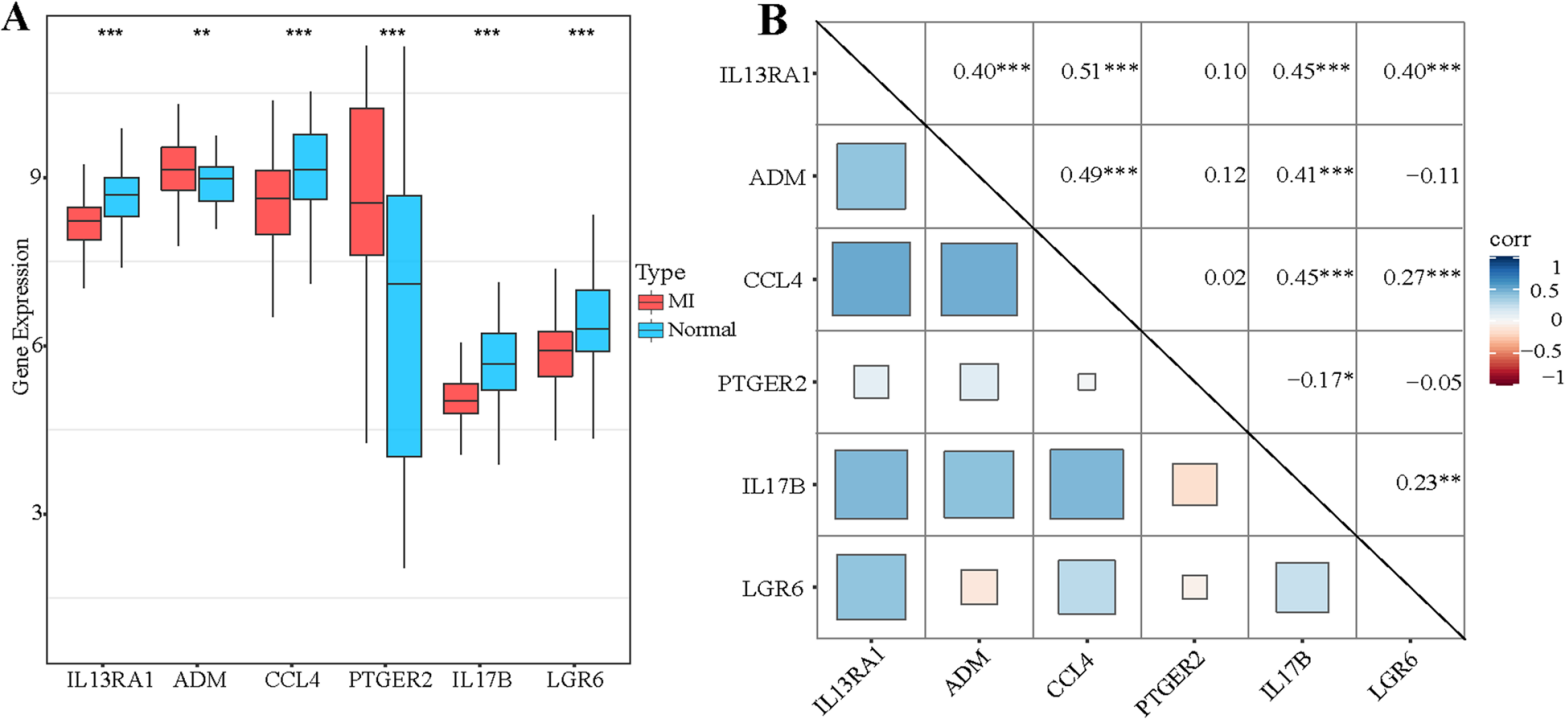
**A** Relative expression level of PTGER2, LGR6, IL17B, IL13RA1, CCL4, and ADM. **B** Correlation among PTGER2, LGR6, IL17B, IL13RA1, CCL4 and ADM

The six most vital explanatory variables (PTGER2, LGR6, IL17B, IL13RA1, CCL4, and ADM) were selected for further analysis. The expression of PTGER2 and ADM in MI patients was more highly expressed than in healthy patients, while the other four genes (LGR6, IL17B, IL13RA1, and CCL4) were low expressed in MI patients (Fig. 5A). The correlations of those genes are analyzed in Fig. 5B, in which IL13RA1 was found to be positively related to LGR6, IL17B, CCL4, and ADM, and IL17B was positively related to ADM and CCL4.

### A nomogram model constructed and assessed for diagnosing MI

**Fig. 6:**
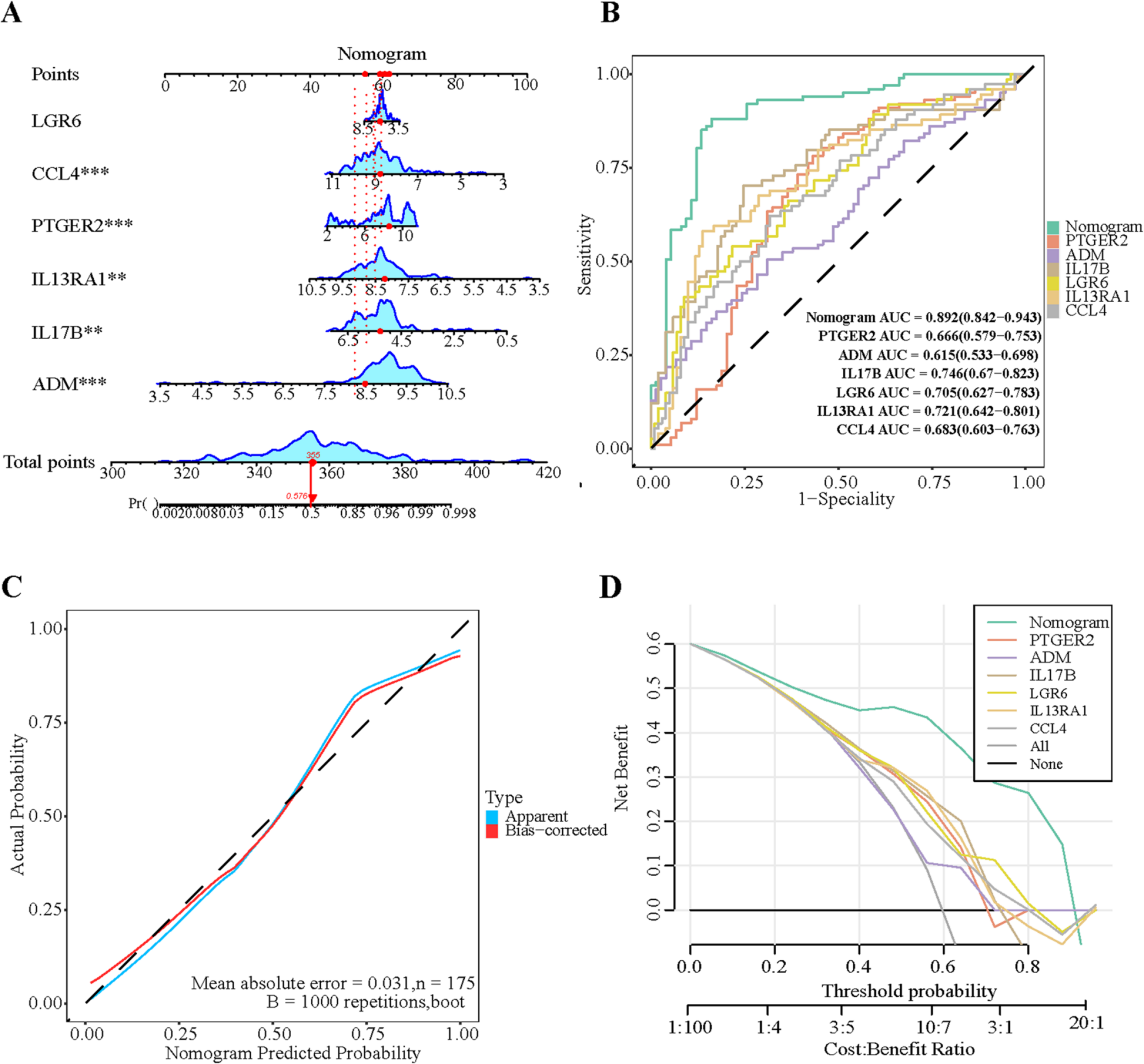
Validation and assessment of a nomogram model for MI diagnosis. **A** Nomogram model. **B** The AUC of the nomogram model in predicting the incidence of MI. **C** Calibration curve to assess the predictive value. **D** DCA curve to evaluate the clinical value

The nomogram model was implemented for diagnosing MI based on six DIRGs (PTGER2, LGR6, IL17B, IL13RA1, CCL4, and ADM) using the rms package in R (Fig. 6A). The nomogram constructed by multivariable logistic regression.The result of multivariable logistic regression was shown in Table 2. Independent risk factors for incidence of MI included PTGER2 gene (OR:1.495,95%:1.239–1.866, *p* < 0.001), ADM gene (OR:5.817,95%:2.955–12.649, *p* < 0.001), IL17B gene (OR:0.322,95%:0.158–0.615, *p* = 0.001), IL13RA1 gene (OR:0.245,95%:0.098–0.562, *p* = 0.001), and CCL4 gene (OR:0.399,95%:0.226–0.673, *p* = 0.001). The Area Under Curve(AUC) was implemented to assess the discrimination ability of the nomogram model, which exhibited the highest predictive accuracy compared with the six above DIRGs (Fig. 6B), while the calibration curve demonstrated a small error between the actual MI incidence and predicted incidence (Fig. 6C). The decision curve (DCA) demonstrated that the nomogram model exhibited better potential clinical utility than the other curves, indicating that patients with MI can benefit from the nomogram model at high-risk threshold probabilities ranging from 0 to 0.8 (Fig. 6D).

**Table 2:** Results of multivariable logistic regression

	OR	2.50%	97.5%	95%CI	*P* value
PTGER2	1.495	1.239	1.866	1.495(1.239–1.866)	*p* < 0.001
ADM	5.817	2.955	12.649	5.817(2.955–12.649)	*p* < 0.001
IL17B	0.322	0.158	0.615	0.322(0.158–0.615)	0.001
LGR6	0.739	0.418	1.276	0.739(0.418–1.276)	0.282
IL13RA1	0.245	0.098	0.562	0.245(0.098–0.562)	0.001
CCL4	0.399	0.226	0.673	0.399(0.226–0.673)	0.001

### Distribution of immune cells in MI

**Fig. 7:**
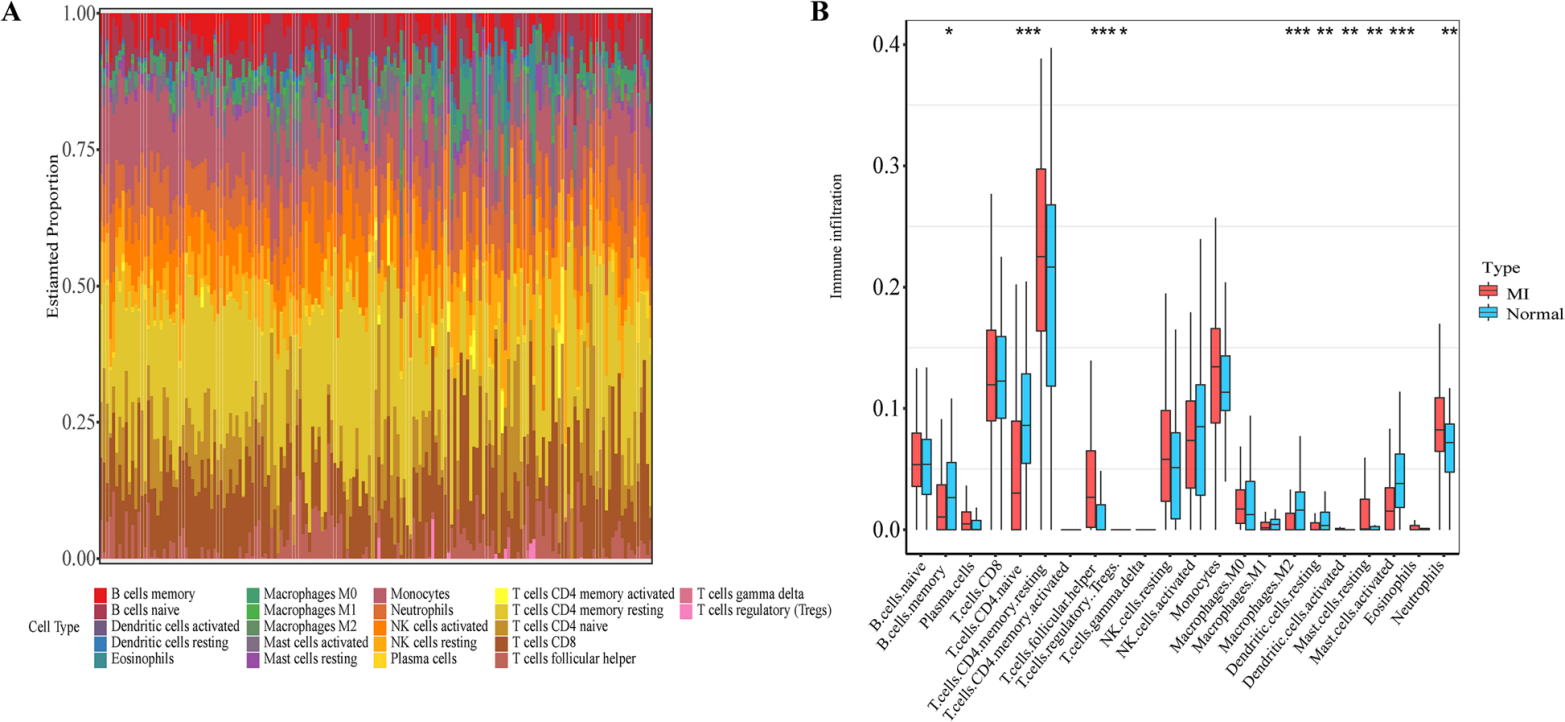
**A** Distribution of the immune cells of all samples. **B** Different distribution of 22 immune cells between patients with MI and healthy controls

To explore the association between immune cell infiltration and the incidence of MI, the 22 types of immune cell infiltration in each sample were calculated by CIBERSORT and visualized by histogram (Fig. 7A). Twenty-two immune cell infiltration between MI patients and normal controls were visualized by boxplot (Fig. 7B). We found that plasma cells, T cells follicular helper, mast cells resting, and neutrophils were higher in MI patients than in healthy patients, while the T cells CD4 naive, macrophages M1, macrophages M2, dendritic cells resting, and mast cells activated in MI patients were lower.

### The association between diagnostic DIRGs and infiltrating immune cells of MI

**Fig. 8:**
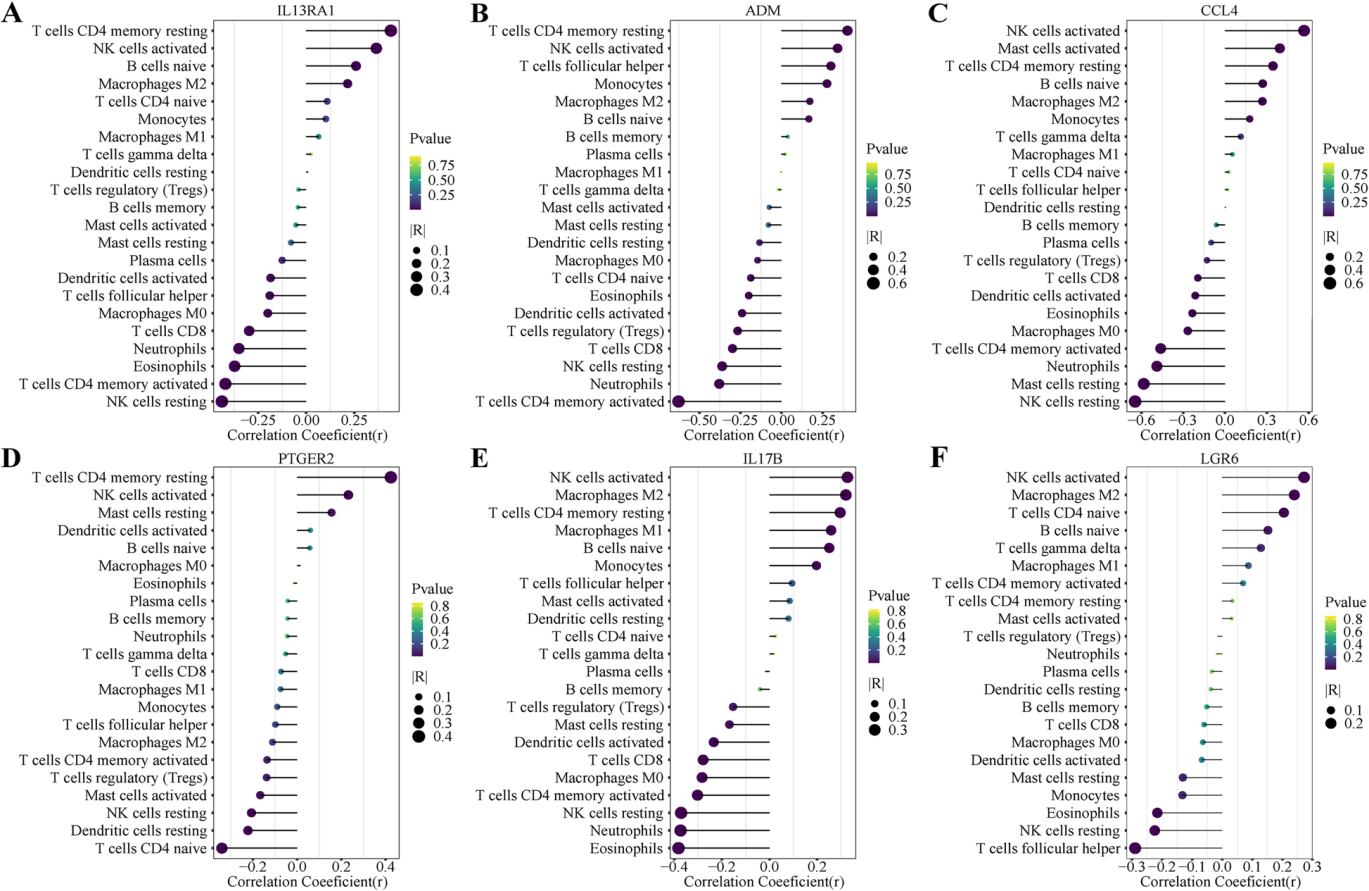
Correlation between IL13RA1 (**A**), ADM (**B**), CCL4 (**C**), PTGER2 (**D**), IL17B (**E**), LGR6 (**F**) and infiltrating immune cells in myocardial infarction

The IL13RA1 gene was positively related to T cell CD4 memory resting. The ADM gene was positively related to T cell CD4 memory resting. The CCL4 gene was negatively related to Natural Killer (NK) cell resting. The PTGER2 gene was positively correlated with T cell CD4 memory resting. The IL17B gene was negatively correlated with neutrophils. The LGR6 gene was negatively correlated with T cells and the follicular helper (Fig. 8).

## Discussion

MI, which is considered one of the most severe complications of CAD, is the main cause of mortality globally [1]. The short-term mortality of patients with MI can be improved by early diagnosis through appropriate medical therapy and revascularization blockage of the coronary artery [2]. Infarction of cardiomyocytes can lead to left ventricular dilatation dysfunction, which eventually contributes to heart failure with high long-term mortality after MI [24]. MI is strongly related to the immune system, but the underlying mechanism of inflammatory mediators in the progress of MI is poorly understood. Thus, the role of inflammatory mediators has been investigated using machine learning. A nomogram model was constructed for the diagnosis of MI based on the DIRGs.

Injured cardiomyocytes can secrete damage-associated molecular patterns (DAMPs) containing heat-shock proteins and mitochondrial DNA [25], which can initiate the immune response by recognizing and linking to extracellular or intracellular pattern recognition receptors (PRRs) on immune cells [24]. However, the role of other inflammatory mediators remains unknown in MI. In our study, 58 DIRGs were enriched in the GO, KEGG, and DO pathway analysis. The six key IRGs (PTGER2, LGR6, IL17B, IL13RA1, CCL4, and ADM) were selected by four machine learning methods, such as PLS, RF, KNN, and SVM, and were constructed for further analysis.

PTGER2, which encodes a receptor for prostaglandin E2, can increase intracellular cAMP concentration and initiate smooth muscle cell relaxation [26]. Almudena reported that PTGER2 was significantly expressed in atherosclerotic plaques in humans. The chemokine production of human macrophages was suppressed by PGE2 through PTGER2 in the progression of atherosclerosis plaque [27]. Leitinger found that, when highly expressed in endothelial cells and monocytes, PTGER2 can induce vascular inflammation and atherogenesis [28]. PTGER2 may be a drug target in atherosclerosis. PTGER2 was upregulated in MI patients compared with normal controls in our study, which was positively related to T cell CD4 memory resting.

LGR6 is a subgroup of the leucine-rich-repeat-containing G protein–coupled receptor (LGR) superfamilies. Ruan et al. reported that LGR6 plays a significant role in the chemoresistance of ovarian cancer by potentiating the Wnt/β-catenin signaling [29]. Chiang et al. reported that LGR6 can enhance phagocytosis and efferocytosis of MΦ and initiate intracellular phosphorylation signaling in neutrophils by binding to maresin 1 [30]. Our study showed that LGR6 was downregulated in MI and that LGR6 was positively related to NK cell activation.

IL-17B is a proinflammatory cytokine. Irez-Carrozzi et al. demonstrated that IL-17B is associated with inflammatory disease, which can trigger type 2 immune responses from NKT, CD4 + CRTH2 + Th2, and innate type 2 lymphocytes (ILC2s) [31]. Zhou et al. found that the expression of IL-17B was significantly highly expressed in patients with community-acquired pneumonia, and IL-17B could upregulate the expression of IL-8 by initiating p38 mitogen-activated protein kinase (MAPK) and extracellular signal-regulated kinase (ERK) in human bronchial epithelial cells [32]. IL-17B was found to be downregulated in MI patients in the current study, and this was found to be positively related to NK cell activation.

IL-13 is a cytokine participating in normal immune function. Gwiggner et al. suggested that IL-13 was secreted by activated type 2 T helper (Th2) cells. The mechanism of IL-13 signaling, here via binding to IL-13 receptor α-1 and IL-13 receptor α-2, started by initiating phosphorylation of the signal transducer and activating transcription 6 (STAT6) via Janus kinases (JAK) [33]. Amit et al. reported that IL-13RA1 participated in the homeostasis and repair of the myocardium. IL-13RA1 signaling was significant for cardiac, including extracellular matrix integrity. Stimulation of IL-13RA1/STAT3 signaling can induce the excessive accumulation of included extracellular matrix, cardiac fibrosis, chronic cardiac stress, and heart failure [34]. The IL-13RA1 was found to be downregulated in MI patients in the current study, and IL-13RA1 was positively associated with T cell CD4 memory resting.

CCL4 is a significant chemotactic mediator for recruiting macrophages [35]. CCL4 is related to the pathogenesis of several diseases, including sarcoidosis [36], cystic fibrosis [37], and multiple sclerosis [38]. Kalinskaya et al. reported that non-ST-elevation myocardial infarction (NSTEMI) patients demonstrated significantly higher expression of CCL4 compared with ST-elevation myocardial infarction (STEMI) patients. The synergy of TNF-a and CCL4 in STEMI patients can be lowly expressed in monocytes that are mediated and increased through the adhesion of leukocytes by TNF-a [39]. CCL4 was downregulated in MI patients in the current study and was positively associated with NK cell activation.

Various tissues, such as the myocardium, adrenal medulla, and central nervous system, can secrete ADM [40]. ADM plays a prominent role in vasodilation, stimulation of angiogenesis, and the production of NO [41]. Ali reported that the ADM expression levels were highly elevated in the plasma of hypertension patients [42]. Previous studies have demonstrated that the vasodilation effects of ADM were mediated by cyclic adenosine 3,5-monophosphate and nitric-oxide-dependent mechanisms. ADM can regulate myocardial protection by disrupting mitochondrial metabolism and lowering the renin-aldosterone system levels in cardiovascular diseases [43]. The ADM was highly regulated in MI patients in the current study and positively associated with T cell CD4 memory resting.

Acute myocardial infarction (AMI) is generally diagnosed by typical symptoms, electrocardiographic changes, and traditional biomarkers, such as hs-cTnT,hs-cTnI, and creatine kinase-MB,but traditional biomarkers have been demonstrated with high sensitivity but without specificity. Previous studies have shown that ncRNAs appears to provide better sensitivity and specificity in diagnosis of MI. MiR-1 participates in regulation of cardiac development and differentiation of other cell types to cardiomyocytes [44]. miR-1 was highly correlated with cTnT in MI patients and demonstrated with a high specificity value (0.82), and sensitivity value (0.73) [45]. miR-499 plays a significant role in cardiac cell recovery and stem cell differentiation [46]. miR-499 had the most accurate predictive value (AUC of 0.91, sensitivity of 0.83, and specificity of 0.90) in distinguishing MI and control [47]. LncRNA H19 had the relatively high predictive value (AUC of 0.753, sensitivity of 0.609, and specificity of 0.817) in MI patients and healthy control,and positively correlated with troponinT (*r* = 0.344, *p* < 0.001), CK(*r* = 0.261, *p* = 0.001) and CKMB (*r* = 0.24, *p* = 0.002) [47, 48]. Compared to ncRNAs, the six DIRGs had shown the relatively high predictive value, and the nomogram model exhibited the highest predictive accuracy (AUC of 0.892, sensitivity of 0.881, and specificity of 0.838) in my study. The nomogram model was constructed for MI diagnosis using PTGER2, LGR6, IL17B, IL13RA1, CCL4, and ADM. The model had the highest predictive accuracy to distinguish MI and normal patients, which could be applied to identify high-risk groups from 0 to 1(0 for normal,1 for MI).

The distribution of 22 immune cells in patients with MI and normal controls was calculated using CIBERSORT. The ratio of T cells follicular helper, mast cells resting, and neutrophils was higher in MI patients, while the ratio of T cells CD4 naive, macrophages M1, macrophages M2, dendritic cells resting, and mast cells activated was lower. The correlation analysis between six key IRGs (PTGER2, LGR6, IL17B, IL13RA1, CCL4, and ADM) and immune cells all were shown to be associated with T cell CD4 memory resting, NK cells activated, and macrophages M2. Admittedly, inflammatory mediators, such as IRGs and immune cells, have been shown to play an important role in the progression of MI. Intercellular adhesion molecule 1 was involved in lymphocyte migration into the intima. T lymphocytes were found to activate to secrete various cytokines that interact with macrophages. T lymphocytes and macrophages can be activated by the engagement of CD40/CD40L to initiate the production of tissue factor and cytokines that enhance the body’s inflammatory responses [49].

Ortega-Rodríguez reported that the total level of NK cells was elevated in MI patients compared with normal controls. The various phenotypes of NK cells play a different role in the progress of MI. The level of natural killer group 2, member D(NKG2D) + NK cells in peripheral blood tends to decrease in MI patients, which may demonstrate that NKG2D cells migrate to the injured myocardium [50]. The other phenotypes of NK cells mobilize from the myocardium to the peripheral blood to mediate the inflammatory response. The evidence demonstrated that several types of infiltrating immune cells play significant roles in the progress of MI and can be the focus of future therapeutic targets [51].

There are also some limitations to our study. First, the sample size of the merged datasets of MI patients and normal controls was relatively small. Second, the expression of PTGER2, LGR6, IL17B, IL13RA1, CCL4, and ADM should be validated in datasets with a larger sample size by quantitative polymerase chain reaction or Western blot. Third, the characteristics of clinical information did not exist in the GEO datasets, and the predictive value of the nomogram model and traditional biomarkers in the diagnosis of MI could not be compared. Finally, immune cells inferred from the DIRGs may have a prognostic utility in the clinic to identify high risk MI patients, although this needs to be validated in larger cohorts.

## Conclusion

The six IRGs, such as PTGER2, LGR6, IL17B, IL13RA1, CCL4, and ADM, were selected by machine learning associated with the occurrence of MI. The nomogram constructed by the six IRGs above was demonstrated as having higher predictive accuracy in the diagnosis of MI. T cell CD4 memory resting, NK cells activated, and macrophages M2 may participate in the advancement of MI.

## Data Availability

The gene datasets in this study including GSE29111,GSE48060,GSE66360,GSE97320, and GSE113079 can be found in Gene Expression Omnibus (GEO, https://www.ncbi.nlm.nih.gov/geo/).
